# T1-Weighted Sodium MRI of the Articulator Cartilage in Osteoarthritis: A Cross Sectional and Longitudinal Study

**DOI:** 10.1371/journal.pone.0073067

**Published:** 2013-08-05

**Authors:** Rexford D. Newbould, Sam R. Miller, Neil Upadhyay, Anil W. Rao, Peter Swann, Garry E. Gold, Robin K. Strachan, Paul M. Matthews, Peter C. Taylor, Andrew P. Brown

**Affiliations:** 1 Imanova Centre for Imaging Sciences, Hammersmith Hospital, Imperial College London, London, United Kingdom; 2 GlaxoSmithKline, Brentford, United Kingdom; 3 Department of Imaging, Charing Cross Hospital, Imperial College London, London, United Kingdom; 4 Department of Medicine, University of Oxford Medical School, Oxford, United Kingdom; 5 Department of Radiology, Stanford University, Stanford, California, United States of America; 6 Department of Orthopaedic Surgery, Charing Cross Hospital, Imperial College London, London, United Kingdom; 7 Centre for Clinical Neurosciences, Department of Medicine, Imperial College, London, United Kingdom; 8 Kennedy Institute of Rheumatology, Nuffield Department of Orthopaedics, Rheumatology and Musculoskeletal Sciences, University of Oxford, Oxford, United Kingdom; University of Washington School of Medicine, United States of America

## Abstract

Structural magnetic resonance imaging (MRI) has shown great utility in diagnosing soft tissue burden in osteoarthritis (OA), though MRI measures of cartilage integrity have proven more elusive. Sodium MRI can reflect the proteoglycan content of cartilage; however, it requires specialized hardware, acquisition sequences, and long imaging times. This study was designed to assess the potential of a clinically feasible sodium MRI acquisition to detect differences in the knee cartilage of subjects with OA versus healthy controls (HC), and to determine whether longitudinal changes in sodium content are observed at 3 and 6 months. 28 subjects with primary knee OA and 19 HC subjects age and gender matched were enrolled in this ethically-approved study. At baseline, 3 and 6 months subjects underwent structural MRI and a 0.4ms echo time 3D T1-weighted sodium scan as well as the knee injury and osteoarthritis outcome score (KOOS) and knee pain by visual analogue score (VAS). A standing radiograph of the knee was taken for Kellgren-Lawrence (K-L) scoring. A blinded reader outlined the cartilage on the structural images which was used to determine median T1-weighted sodium concentrations in each region of interest on the co-registered sodium scans. VAS, K-L, and KOOS all significantly separated the OA and HC groups. OA subjects had higher T1-weighted sodium concentrations, most strongly observed in the lateral tibial, lateral femoral and medial patella ROIs. There were no significant changes in cartilage volume or sodium concentration over 6 months. This study has shown that a clinically-feasible sodium MRI at a moderate 3T field strength and imaging time with fluid attenuation by T1 weighting significantly separated HCs from OA subjects.

## Introduction

Knee osteoarthritis (OA) is a debilitating disease with cartilage degeneration as a major feature of the disease process. The standard assessment of efficacy of OA therapies for regulatory agencies is via radiographs taken over a period of one or more years [1]. There is considerable interest in methodologies which could give a reliable indication of cartilage degradation or preservation over short timescales in order to evaluate new interventions and pharmaceutical therapies. Disappointingly, average cartilage volume change as detected by structural MRI was only 4-6% per year [2], with no consensus on measurement or analysis methodology. MRI measures of cartilage integrity have focused on T2 [3], T1rho [4], and the delayed gadolinium enhancement MRI of cartilage (dGEMRIC) [5]. Interpretation of T2 changes is complicated by sensitivity to collagen degradation, tissue hydration, and fibril orientation [6], while T1rho is known to be sensitive to several macromolecular components [6].

Sodium MRI has shown promise as a technique to image the integrity of articular cartilage in vivo [7,8]. Mobile ions such as sodium will distribute in cartilage in proportion to the proteoglycan concentration, relatively unaffected by the sodium content or inflammation of the synovium [9]. In cartilage samples, sodium imaging has been shown to be sensitive to small changes in proteoglycan concentration [7,8]. Unfortunately, sodium MRI requires specialized hardware and software, and the low innate signal to noise ratio (SNR) of sodium MRI demands long imaging times. Current sodium MRI methods [10–12] have focused on sodium-density weighted imaging, requiring long TRs in order to assure full T1 recovery of all species. Further, inversion-recovery (IR) based sodium MRI has now been explored to remove the synovial fluid component [13–15] which can affect the measured cartilage sodium concentration by the partial voluming effect of the low resolution achievable in sodium MRI. The major drawback of these IR methods are a 40% loss of SNR [15], or equivalently half the SNR efficiency [16], which requires twice the scan time in order to recover the lost SNR.

As the sodium T1 at 3T of synovial fluid is around 60ms but only 18ms in cartilage [8,17,18], a T1-weighted short TR sequence would preferentially attenuate the fluid signal over the cartilage signal. Furthermore, the SNR per unit time could be increased by up to 50% [19]. To this date, no study has examined the ability of T1-weighted sodium MRI to map longitudinal and cross-sectional differences in the knee cartilage in OA. Therefore, the aim of the study was to assess differences at baseline in the sodium content of the knee cartilage of OA subjects (OAs) to that of healthy controls (HCs) matched for age and gender as determined by T1-weighted sodium MRI. A further aim was to determine whether changes in cartilage integrity, as indicated by changes in sodium content, are observed at 3 and 6 months in order to determine the utility of sodium MRI as an endpoint for clinical drug trials.

## Methods

### Patient Population

A total of 31 OA subjects with documented diagnosis of knee OA meeting the 1986 American College of Rheumatology (ACR) criteria for primary knee OA [20] were recruited from rheumatology and surgical clinics within Imperial College NHS trusts hospitals into the OA cohort. Three subjects withdrew after screening and enrollment but before any imaging sessions, resulting in 28 complete datasets in the OA group. A total of 23 healthy subjects were also recruited into the HC cohort, each healthy subject recruited to age (± 5 years) and gender match an OA subject. Four subjects withdrew in the HC group, resulting in 19 complete datasets in healthy subjects. Healthy subjects were defined as individuals who were free from clinically significant illness or disease as determined by their full medical history (including family), physical examination, and previous laboratory studies. Sample sizes were determined based on the effect size seen in previous small cohort studies [17]. All subjects were recruited and gave written informed consent in accordance with a research ethics committee approved (Redbridge and Waltham Forest REC ref: 08/H0701/87) study protocol.

All subjects attended the baseline visit within 28 days of the screening visit, followed by visits at 3 months and 6 months post-baseline. During each visit subjects underwent clinical assessments including a pain visual-analog scale (VAS) score, the International Physical Activity score (IPAQ) [21], and the Knee injury and Osteoarthritis Outcome Score (KOOS) [22]. Following this, subjects had a single MRI scanning session. All subjects also had standing plain film x-rays taken at the baseline visit only, and whole joint Kellgren-Lawrence scores [23] were derived by a radiologist with 16 years’ experience, blinded to the subjects’ cohort.

### Phantom Imaging

A range of sodium gel phantoms were created in 50mL plastic tubes. Five 10% by weight agar phantoms were constructed with 150, 200, 250, 300, and 350mM sodium concentration to mimic healthy cartilage [8]. In addition, 300mM sodium phantoms were also constructed with 4%, 6%, and 8% by weight agar to mimic partially degraded cartilage, which may be expected to have longer sodium T1 and T2 relaxation times [18] [8]. Finally, 0% by weight agar (saline) phantoms were constructed with a stronger 250mM concentration as well as 150mM sodium to mimic synovial fluid.

### MR Imaging

Phantoms and subjects were scanned in a Siemens 3T Tim, a Trio System (Siemens Healthcare, Erlangen, Germany) MRI scanner in all three visits with an 18cm diameter dual tuned proton-sodium quadrature volume coil (Rapid Biomedical GmbH, Rimpar, Germany) positioned at the magnet’s isocentre.

Sodium MRI images were acquired using an ultra-short TE 3D cones [11,24] readout. 712 3D cones readouts of 1052 readout points were acquired per volume for a final isotropic resolution of 2.5mm in an 18cm FOV, TE=270µs, and a nominal flip angle of 70°, with 118 averages. Subjects were imaged with a T1-weighted acquisition using a TR of 15ms, requiring 21 minutes. Phantom experiments used an identical setup, but were imaged with a range of TRs: 15ms, 30ms, 50ms, and 100ms, requiring 21, 42, 70, and 140 minutes, respectively.

High resolution 3D structural dual-echo steady state (DESS) [25] was acquired at 600µm isotropic resolution in 6m: 34s in 160 slices in a 3D sagittal slab with 15cm FOV using a TR of 14.84ms, TE of 5.04ms, and a 25° water-excitation RF pulse. Structural scans were also acquired at baseline and were reviewed to exclude other knee pathologies. Two of the phantoms with 150 and 250mM sodium concentration were included in the field of view for all scans for signal normalization.

Sodium images, [Na], were corrected for partial recovery of the sodium signal using the median signal in the ROI covering, T1 of, and known concentration in the stronger of the two reference vials in the in-vivo images and the 250mM 10% agar vial in the phantom images, *S*_*VIAL*_, T1_vial_, and [Vial], respectively, as well as a literature T1 value for sodium in the cartilage, T1_*CART*_, at 3T of 18ms [8,17,18] using:

### ROI Definition

The cartilage was segmented on the 3D ^1^H DESS images by a blinded reader into three large cartilage zones: tibial, femoral and patellar. Each region was sub-divided into medial and lateral zones: medial-tibial (MT), lateral-tibial (LT), medial-femoral (MF), lateral-femoral (LF), medial-patellar (MP) and lateral-patellar (LP). The tibial and femoral cartilage was divided into medial and lateral using the midline of the intratrochanteric fossa. The patellar cartilage was split by a vertical line halfway between the medial border of the lateral femoral condyle and the lateral border of the medial femoral condyle. Volumes for each ROI were generated for each timepoint. The sodium MR image was co-registered using the rigid-body registration in spm8 (FIL, Institute of Neurology, London, UK) to correct for any patient movement between the scans. Median sodium concentrations in each ROI were calculated for each sodium scan.

In phantom experiments, ROIs were drawn in each sodium phantom, and the mean over each ROI was reported.

### Statistical Analysis

Repeated measures ANOVA was used on all MRI measures. Separate analyses were run for the volume and median Na concentration for the three different levels of ROIs (whole knee, 3 ROIs, 6 ROIs) making a total of six models. Within each model fixed effect terms were included for group, visit and ROI (aside from the whole knee analysis) and all corresponding 2- and 3-way interactions. Random effects were included for subject and matched-pair. Where applicable the ROIs were considered a repeated measure within each visit with an unstructured correlation matrix. The overall statistical significance of variation between levels of fixed effects (main effects and interactions) was assessed using an F-test applying the Kenward-Rogers correction to the denominator degrees of freedom. Where applicable the statistical significance of pairwise differences between levels was assessed using a t-test. Spearman’s rank correlation coefficients between the primary and secondary endpoints were estimated with 95% confidence intervals (CIs). A non-parametric measure of association was used to avoid assumptions of linear relationships. The Student’s t-test was used to analyze group differences in body mass index (BMI), height, weight, and KOOS scores. Gender was compared with Fisher’s exact test, and the Wilcoxon signed-rank test was used on K-L scores.

## Results

### Clinical Measures

Baseline comparisons between groups are reported in Table 1. The two groups are almost perfectly age-matched, although the protocol allowed a per-subject deviation of up to 5 years. The OA group was highly significantly separated (p=0.0016) from the HC group by KL score, a well-established method of assessing joint degradation radiographically. KOOS and pain scoring systems were still more discriminating between groups (p<0.0001), which may be expected as pain is one of the ACR criteria for OA diagnosis. The IPAQ scoring did not achieve significance (defined as p<0.05), possibly as it only reflects activity levels. The OA group was heavier than the HC group, with a mean BMI of 29.6 versus 24.9 for the HC group. This may be a result of limited mobility due to knee OA.

**Table 1:** Summary of the clinical measures in the two subject groups at baseline.

	OA Subjects						Healthy Controls							
	**n**	**mean**	**SD**	**min**	**max**		**N**	**mean**	**SD**	**min**		**Max**		**p-value**
Age	28	63	9	43	81		19	62	7		50	72	0.93	
Height (cm)	28	162.7	9.4	150	186		19	166.0	7.8		155	178	0.195	
Weight (kg)	28	78.0	15.1	53.5	100		19	68.9	10.2		49.5	85.5	**0.017**	
BMI	28	29.6	5.8	19.2	37.7		19	24.9	2.5		19.8	28.3	**0.0005**	
K-L score	27	2.1	1.2	0	4		18	0.6	0.7		0	2	**0.0016**	
KOOS	28	129	30	76	189		19	39	14		0	53	**<0.0001**	
IPAQ	28	2.6	1	1	3		19	2.8	0		2	3	0.10	
Pain VAS	28	7.3	3	0	10		19	0.3	1		0	3	**<0.0001**	

Among the pair-wise associations between subjects’ clinical assessments, four of the measures had rank correlations (r) ≥ 0.3. Age was correlated with K-L score but did not achieve significance (r=0.30, p=0.096). However, KOOS did achieve significance with K-L score (r=0.51, p=0.0028), as did the pain VAS (r=0.43, p=0.014). KOOS and the pain VAS were well correlated (r=0.89, p<0.0001), which could be anticipated given that pain scores make up part of the KOOS index.

### MRI Measures

**Figure 1:**
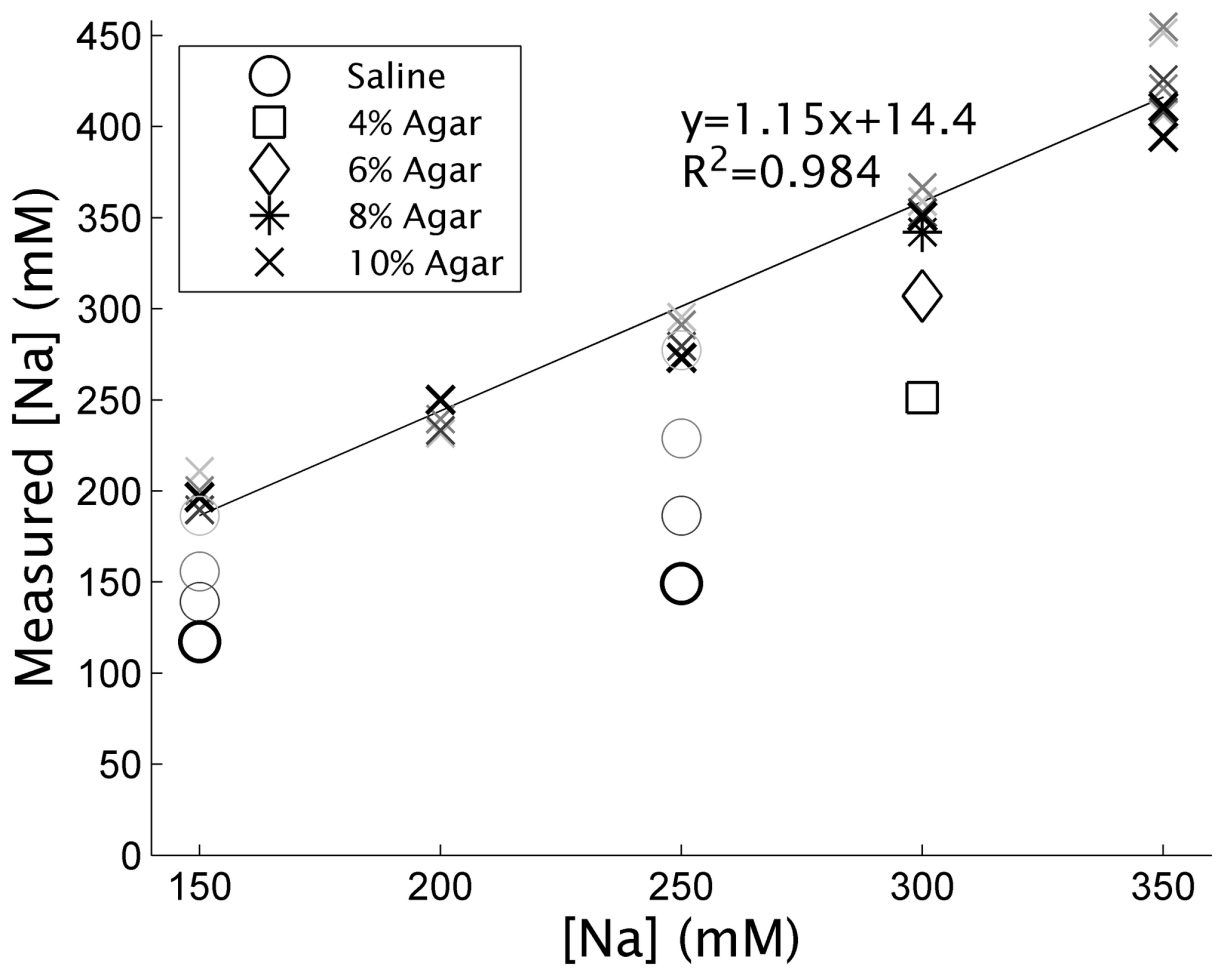
Sodium concentrations measured with 3D UTE imaging of sodium phantoms across varying TRs. The lightest plot symbols correspond to a 100ms TR, darkening with as drops to 50ms, 30ms, and finally 15ms for the darkest plot symbols. All data has been corrected for the incomplete T1 relaxation of cartilage sodium using the 250mM cartilage-mimicking 10% agar phantom. A strong linear relationship between image intensity and mean sodium concentration is found in the 10% agar cartilage like phantoms. Saline ROIs show an underestimation of the sodium concentration, which reduces as TR increases. Lower concentrations of agar underestimate the sodium concentrations more like saline, as could be expected.

Figure 1 displays the results of the phantom imaging experiments. Each phantom vial has four plot marks, one for each TR used (15, 30, 50, and 100ms) in lightening colors with lengthening TR. The uncorrected signal level in the cartilage-mimicking 10% agar solution vials follows a linear trend versus known sodium concentration (R^2^=0.984) regardless of TR used. However, the signal level, and thus measured sodium concentration, of the synovial fluid-mimicking saline solutions (circles in Figure 1) is depressed. The 15ms TR in the darkest plot symbols, as used in the clinical imaging, underestimates the sodium concentration in saline by almost 50%. Synovial fluid has a lower sodium concentration of 150mM versus the concentration in healthy cartilage of 250 to 300mM [8,26]. Therefore, in the clinical imaging, synovial fluid sodium signal intensity would be very hypointense. The decreasing agar concentrations increased the underestimation of the sodium signal, as expected. The 8% agar solution of 300mM sodium underestimates the sodium concentration by only 4.25% versus the 10% solution, whereas the 4% solution underestimates by 35%.

**Figure 2:**
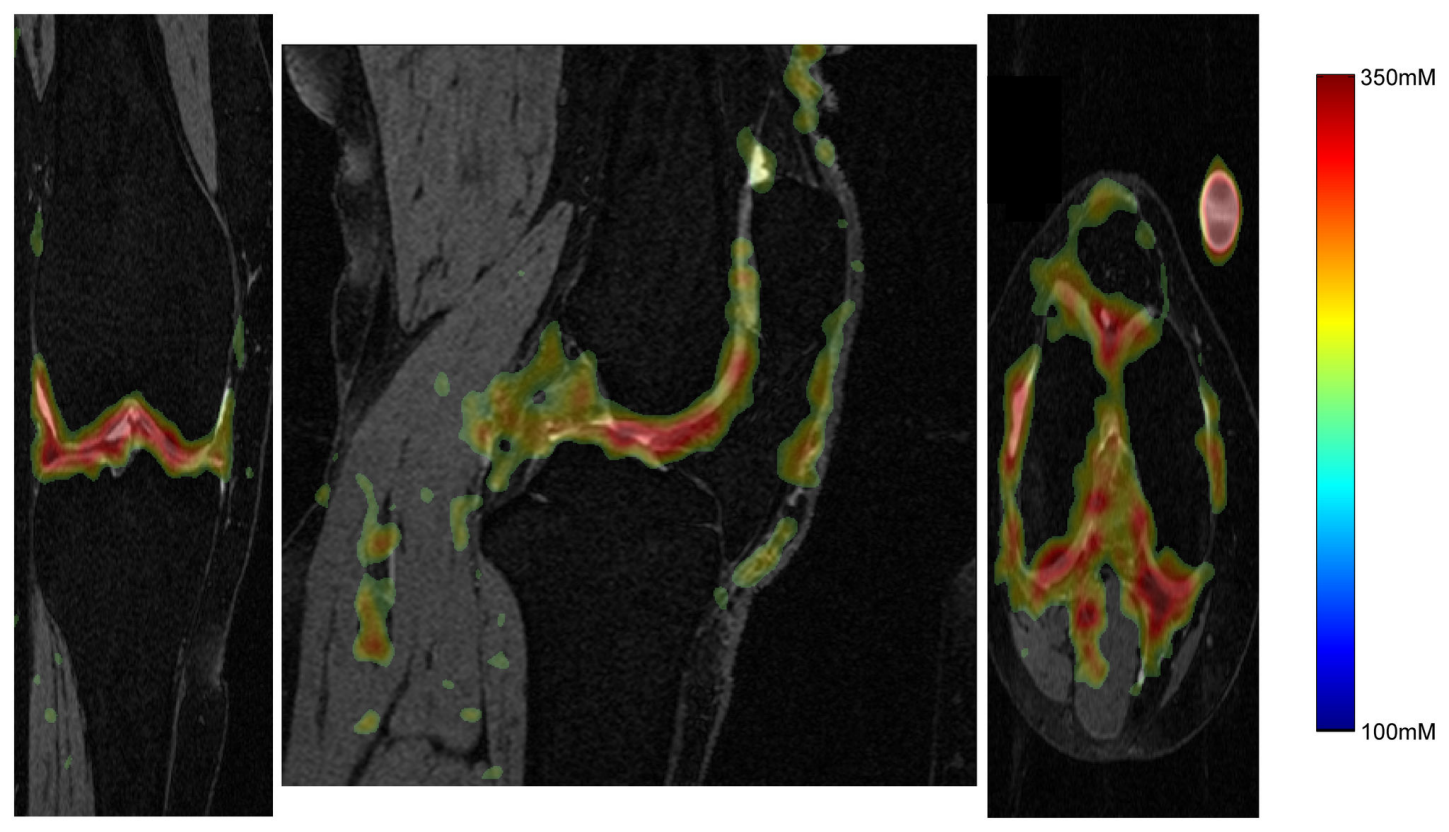
Example 3D sodium concentration dataset overlaid on the 3D DESS image. High values of sodium concentration can be found co-localized with the articular cartilage.

Figure 2 demonstrates the 3D UTE T1-weighted sodium concentration imaging overlaid on the 3D DESS image for an OA subject. High sodium concentration (red) can be appreciated in the cartilage. Table 2 reports the average values for volume and median Na concentration with standard errors found across each cohort for each of the three visits. Values are first reported for the whole-cartilage ROI, and then subdivided into the femoral, patellar, and tibial regions, before the medial and lateral subdivisions of the three regions. Table 3 summarizes the statistically significant (p<0.05) findings from Table 2 with the six ANOVA models. There was a 20% smaller patellar cartilage volume in OA subjects compared to healthy controls, which was strongly significant. The femoral cartilage was slightly (8.2%) larger in OA subjects, but this did not reach significance. As expected from prior work, there were no significant changes in volume with time for either group over this 6 month period.

**Table 2:** Volume and Sodium Concentrations for each ROI for all 3 visits in each cohort.

	**GROUP**	**VISIT**	**WHOLE**	**FEM**	**PAT**	**TIB**	**LF**	**MF**	**LP**	**MP**	**LT**	**MT**
Volume		1	19.1 (1.0)	11.5 (0.7)	3.1 (0.2)	4.6 (0.3)	5.9 (0.4)	5.6 (0.4)	1.9 (0.1)	1.2 (0.1)	2.5 (0.1)	2.1 (0.1)
	HC	2	18.6 (1.0)	11.2 (0.7)	3.1 (0.2)	4.4 (0.3)	5.8 (0.4)	5.5 (0.4)	1.9 (0.1)	1.3 (0.1)	2.4 (0.1)	2.1 (0.1)
		3	18.4 (1.0)	11.0 (0.7)	3.0 (0.2)	4.4 (0.2)	5.7 (0.3)	5.3 (0.4)	1.8 (0.1)	1.2 (0.1)	2.4 (0.1)	2.0 (0.1)
		1	19.4 (0.9)	12.6 (0.6)	2.4 (0.1)	4.7 (0.2)	6.3 (0.3)	6.3 (0.4)	1.4 (0.1)	0.9 (0.1)	2.5 (0.1)	2.3 (0.1)
	OA	2	19.5 (0.9)	12.1 (0.7)	2.5 (0.1)	4.5 (0.2)	6.3 (0.3)	5.8 (0.4)	1.6 (0.1)	0.9 (0.1)	2.3 (0.1)	2.2 (0.1)
		3	19.3 (0.9)	12.0 (0.6)	2.5 (0.1)	4.5 (0.2)	6.1 (0.3)	5.9 (0.4)	1.6 (0.1)	1.0 (0.1)	2.3 (0.1)	2.2 (0.1)
[Na]												
		1	318 (12)	258 (14)	307 (12)	310 (13)	330 (12)	267 (16)	250 (13)	295 (14)	321 (12)	318 (12)
	HC	2	326 (12)	265 (14)	321 (12)	318 (13)	334 (12)	272 (16)	256 (13)	312 (14)	336 (12)	326 (12)
		3	316 (12)	257 (13)	321 (11)	309 (13)	328 (12)	260 (15)	252 (12)	309 (14)	340 (12)	316 (12)
		1	343 (11)	281 (13)	345 (11)	347 (12)	340 (11)	279 (14)	282 (11)	355 (13)	337 (11)	343 (11)
	OA	2	349 (11)	284 (13)	344 (11)	361 (13)	344 (11)	289 (15)	283 (12)	365 (14)	335 (11)	349 (11)
		3	332 (11)	286 (13)	340 (11)	341 (12)	326 (11)	293 (14)	277 (11)	360 (13)	335 (11)	332 (11)

Values reported are the mean (standard error) across subjects.

**Table 3:** Summary of statistically significant (p<0.05) findings from repeated measures ANOVA models within each ROI.

	**Effect**	**Whole knee**	**Fem/Pat/Tib**	**LF/LP/LT/MF/MP/MT**
Volume	Group	-	OA<HC in Pat (p=0.0029)	OA<HC in LP (p=0.0046), MP (p=0.0065)
	Visit	-	-	-
[Na]	ROI	-	{Fem,Tib} > Pat (p<0.0001)	{LF,MF,LT,MT} > {LP,MP} (p<0.0001)
	Group	OA>HC (p=0.019)	OA>HC in Fem (p=0.038) Pat (p=0.034) Tib (p=0.014)	OA>HC in LF (p=0.0014), LT (p<0.0001), MP (p=0.0093)
	Visit	-	-	-

denotes no significant findings

**Figure 3:**
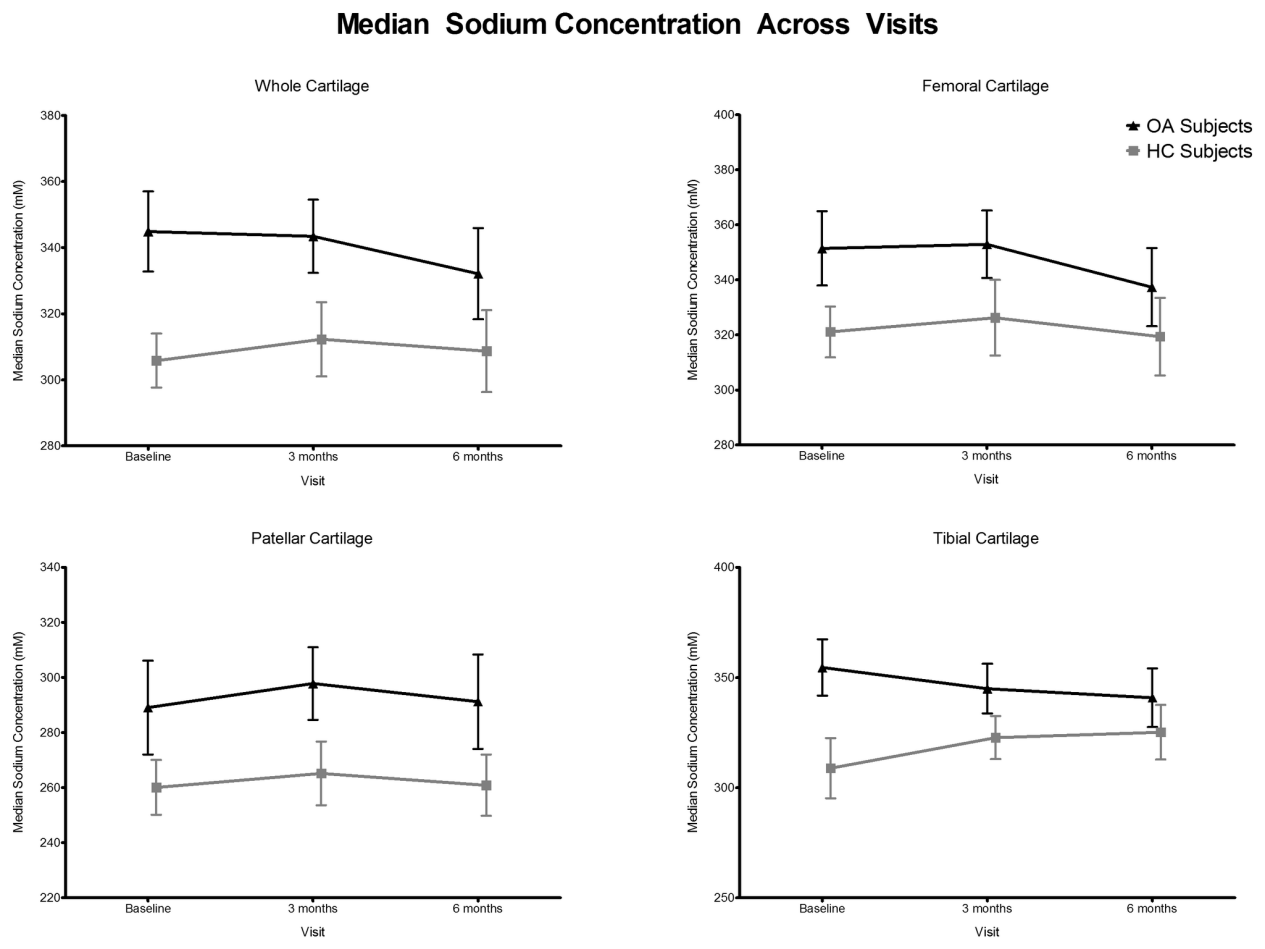
Group means in OA and HC subjects of median sodium concentration at baseline, 3 months and 6 months for whole, tibial, femoral and patellar cartilage ROIs. Whiskers denote the standard error of the mean.

Median sodium concentration in the femoral and tibial ROIs was greater than in the patellar ROI by 21%. OA subjects had an 8% higher sodium concentration in the whole knee than HCs, with this increase most strongly observed in the lateral tibial (18% higher), lateral femoral (12% higher) and medial patellar (11% higher) ROIs, all of which were significant. There were no significant changes in concentration over time for OA subjects or HCs. The stability of the sodium concentration across time, as well as the separation between groups, can be appreciated graphically in Figure 3, which plots the average of each group’s sodium concentration at all three visits. At all timepoints, in all 3 ROI regions as well as the whole knee, OA subjects had a stronger sodium concentration than HCs. Medial and lateral delineated ROIs follow similarly (data not shown).

Table 4 summarizes comparisons between the MRI measures of volume and sodium concentration with the clinical scoring metrics. Only correlations with |r|≥0.3 are shown. The patellar ROI’s volume did correlate with pain and KOOS scoring, and the medial patellar volume also correlated with K-L score. The sodium concentration over the entire knee showed a weak relationship (r=0.31) with pain score, as did the lateral tibial and medial patellar sodium concentrations.

**Table 4:** Summary of associations between imaging and clinical measures with |r|≥0.3.

	**Whole knee**	**Fem/Pat/Tib**	**LF/LP/LT/** **MF/MP/MT**
Volume	No significant associations	Pat: KOOS r = −0.46, Pain VAS r = −0.49	LP: KOOS r = −0.52 Pain VAS r = −0.56, MP: Age r = −0.43, K-L score r = −0.45, KOOS r = −0.37, Pain VAS r = −0.38
[Na]	Pain VAS r = 0.31	No significant associations	LT: KOOS r = 0.42 Pain VAS r = 0.44, MP: Pain VAS r = 0.32

## Discussion

This study has shown that T1-weighted sodium MRI can significantly differentiate knee cartilage in subjects with OA from age-matched controls without OA symptoms. Previous pilot studies have compared sodium concentrations, as determined by MRI, in the knee cartilage of OA subjects and healthy controls. However there are a number of major differences to this study. Previous studies concluded that lower sodium values would be found in the diseased cartilage of OA subjects than in healthy cartilage, as had been identified by in-vitro cartilage enzymatic degradation studies [7,18,27]. The seminal study comparing OA and HC subjects’ cartilage in-vivo [17] used 9 HCs with a mean age of 26 (range 21-31 years) versus 3 OA subjects with a mean age of 43 (range 38-50 years). ROIs were specifically drawn in areas of lower sodium signal for the OA subjects and compared against a whole-patellar cartilage ROI for HCs. More recent work at 7T concluded with similar difference in sodium concentration [28]. In that work, both groups contained 5 subjects, though the HC group had a mean age of 28.7 ± 4.8 years compared to the OA group’s mean age of 52.4 ± 5.6 years. Although the study’s stated purpose was not to compare groups, reduced sodium was reported in one OA subject. Regions of depressed signal were not specifically delineated, but an ROI was drawn on the sodium image that bridged the femoral and tibial regions, and thus included synovial fluid between the regions, and histograms were compared. In this study, in contrast to previous studies, the demarcation of ROIs on the sodium image was specifically avoided due to concerns of biasing the results towards areas of lower sodium concentration. ROIs were defined on the independent DESS contrast, and applied to the coregistered sodium images.

Interestingly, it has been shown that age may be an independent factor in the decline of sodium MRI signal. Using an identical setup as the cross-sectional study at 7T [28], another group has compared [29] a group of 3 young (age range 20-24 years) to 3 old (age range 43-59) subjects, and found an average 15% less sodium signal in the older but otherwise healthy group. Therefore, age-matched groups, as used here, would be vital to validating sodium MRI in OA. Age-matched comparisons of sodium MRI in OA have not previously been reported.

Several groups have noted that the suppression of sodium signal from fluids surrounding the cartilage may be important in correctly attributing sodium signal to cartilage. Fluid suppression via inversion recovery in sodium sequences [13] can effectively remove the confounding synovial fluid signal, though at the penalty of reduced SNR in the cartilage signal. The sodium concentration in the fluid is about half that of the cartilage, even so, fluid suppression does result in higher sodium values [16]. The acquisition in this study used a T1-weighted acquisition, which results in preferential fluid suppression due to its much longer T1. The short T1 of sodium in cartilage would result in 28% less signal than using a fully-relaxed (TR > 5*T1) acquisition, but 74% less sodium signal in the synovial fluid. The shorter TRs permit more averaging in the same clinically acceptable scan time, improving SNR. The SNR efficiency at this TR is 43% higher than that at 100ms TR. ROIs in this study were defined on the DESS images and applied to the sodium images. One might expect partial voluming between the cartilage and fluid to be worse in OA subject due to thinner cartilage and/or fluid invasion of the cartilage [30]. This would result in a spuriously lower measured sodium concentration in OA subjects, the opposite of what was found. Furthermore, an increased T1 in the diseased cartilage [18] would also lower the detected sodium concentration, again opposed to the results. The results presented here show between a 10 and 20% increase in measured sodium concentration in the diseased cartilage. If this effect was entirely a change in the underlying sodium T1 value, the sodium T1 of the diseased cartilage would need to decrease by 24 to 33%. Only increases in sodium T1 have been noted with cartilage degradation, however, decreases have been noted in mechanical compression of ex-vivo cartilage samples [31]. In this study, care was taken to ensure all subjects had similar recent activity levels and identical positioning in the MRI system.

The median sodium concentration in the femoral and tibial ROIs was greater than the patellar ROI. This is of interest as the patellar cartilage is the most often studied in sodium MRI of OA. A surface coil placed on the kneecap is the most common acquisition setup. This gives very good SNR near the surface coil, such as in the patellar cartilage, but poor SNR in regions further away, such as the femoral condyles. This study used a volume sodium coil, which results in lower maximum SNR in any one region, but more homogenous transmit and receive RF profiles, reducing the need for their correction, and giving more equal sensitivity to all areas within the coil. Sodium transmit RF mapping performed during study setup indicated very low variation across the volume; therefore no RF mapping was performed in this study. However, all subjects were carefully positioned to ensure the bulk of the knee cartilage was in the center of the coil, as far away as possible from the RF coil rungs, where the RF field often varies.

Clinical measures for the patient population were in line with expectations as clear differences were found between OA subjects and healthy controls in three clinical measures: KOOS, Pain VAS and KL score. The use of age-matched subjects brings concerns about the relative health of the cartilage in knees of subjects in the 60’s. Therefore, to rule out clinically occult radiographic changes, subjects were re-stratified into OA and HC groups based on their KL scores, defining a KL score of 0 or 1 as HC and a KL score of 2 or greater as OA. After re-stratifying, significant separation of the groups remained in all measures that achieved significance with the previous classification, with OAs continuing to have higher sodium concentration in all compartments (data not shown).

There are a number of important limitations to the interpretation of this study. Primarily, no histological sampling of the cartilage was performed, so no comparison between disease state and sodium concentration can be made. The differences in relative changes between these clinical results and previous preclinical or ex-vivo models may reflect inherent limitations of those latter acute models to reproduce key features of the chronic human disease. However, without histological verification, no conclusions can be drawn. As this study employed T1-weighted sodium MRI, rather than density-weighted sodium MRI, the increased sodium concentration detected could not be differentiated from a shortening of the T1. However, previous studies have shown T1 increases with disease, rather than decreases. The 3T magnetic field strength used here limits sensitivity and resolution, possibly contributing to smaller effect sizes than could be achieved from ultra-high field (e.g. 7T) studies. The number of subjects used here was designed to power a 3T study due to the greater availability of 3T scanners. Finally, all ROI’s were drawn by a single blinded reader, therefore no inter-reader statistics can be examined.

## Conclusions

The overall goal of this study was to validate sodium MR imaging measures of cartilage for use in clinical trials of disease-modifying drugs. Previous reports show good reproducibility of 3.2% within-subject coefficient of variation [32] for sodium MRI in the knee cartilage. This study shows clear differences between OA subjects and healthy controls, although no change was seen over the defined timespan of 6 months.

This study has compared age-matched cohorts of OA and healthy control subjects using sodium MRI for the first time. T1-weighted sodium MRI significantly differentiated OA subjects from healthy controls. This difference was stable over the 6 month timeframe. Volumes of the ROIs were also compared, and patellar volume was seen to be lower with strong statistical significance in OA subjects. Volume was not noted to change over the 6 month time frame; however, it was unlikely to be detected over this timespan, as most structural studies use longer timespans [33].

The results of this study can be used to design and power a clinical trial of a disease modifying intervention for OA. It can be seen that sodium MRI on a 3T scanner can significantly differentiate between knee cartilage in healthy controls and that in subjects with previously-diagnosed OA. However, the timescales needed for such a study are longer than 6 months.
